# Supplementary material for: Unusual competition of superconductivity and charge-density-wave state in a compressed topological kagome metal
Source: Nat Commun. 2021 Jun 10;12:3645. doi: 10.1038/s41467-021-23928-w (PMC8192749; doi:10.1038/s41467-021-23928-w)
Supplement: Supplementary file 1 — Supplementary Information [file 41467_2021_23928_MOESM1_ESM.pdf]

Supplementary Information:

Unusual competition of superconductivity and charge-density-wave state in a compressed topological kagome metal

F. H. Yu[1], D. H. Ma[1], W. Z. Zhuo[1], S. Q. Liu[1], X. K. Wen[1], B. Lei[1], J. J. Ying[1*], and X. H. Chen[1,2,3†]

[1]Hefei National Laboratory for Physical Sciences at Microscale and Department of Physics, and CAS Key Laboratory of Strongly-coupled Quantum Matter Physics, University of Science and Technology of China, Hefei, Anhui 230026, China

[2]CAS Center for Excellence in Quantum Information and Quantum Physics, Hefei, Anhui 230026, China

[3]Collaborative Innovation Center of Advanced Microstructures, Nanjing 210093, People's Republic of China

Supplementary Fig.1a shows the temperature dependence of resistivity up to room temperature, for sample 1 with PCC. Supplementary Fig.1b shows the temperature dependence of resistance up to room temperature, for sample 2 with DAC.

Supplementary Fig.2 shows the high-pressure magnetic susceptibility measurement for sample 3 with PCC. The T_c gradually increases with increasing the pressure below P1, consistent with the high-pressure resistivity measurements. With the pressure above P1, the superconducting volume fraction suddenly decreases. Further increasing the pressure, the bulk superconductivity T_c^{M2} emerges below 4 K around 1.1 GPa, while the magnetic susceptibility shows a weak reduction at higher temperature at T_c^{M1} as shown in Supplementary Fig.2b, indicating a filamentary superconductivity.

Supplementary Fig.3 shows the temperature dependence of resistivity under various magnetic fields with pressure of 1.06 GPa for sample 1 with PCC. The superconducting transition is very broad and T_c can be gradually suppressed by increasing the magnetic field.

Supplementary Fig.4 shows the magnetoresistance measured at 10 K under external magnetic field applied along the *c* axis and at various pressures, for sample 1 with PCC. The magnitude of magnetoresistance suddenly decreases above P1. The low-field magnetoresistance evolves from a "V"-shape to "U"-shape at P2. The different MR behavior can be related to the different electronic structure and electron scattering in the different pressure regions. The linear MR at the low field region below P2 can possibly arise from the linear band crossings[1,2] or/and the CDW state[3]. We also performed the MR measurements under various pressures at 1.02 GPa for sample 4 with PCC as shown in Supplementary Fig.5. The T* is suppressed to 49 K in this sample as determined by $d\rho_{xx}/dT$ in Supplementary Fig.5a. We find that the shape of MR suddenly changes from "V"-shape to "U"-shape above T*, similar with the case by tuning using the pressure. Therefore, the observed linear MR is associated with the CDW order.

We performed magnetoresistance measurements with magnetic field up to 14 T at 2 K, at several pressures for sample 4 with PCC and the results are shown in Supplementary Fig.6. Clear Shubnikov-de Haas (SdH) quantum oscillations (QOs) are observed with pressure below P1 as shown in Supplementary Fig.6a. Above P1, the QOs become much weaker and cannot be resolved at pressure of 1.02 GPa. Analyzing the SdH QOs can provide insight on the Fermi surfaces. After subtracting a slowly changed polynomial background, the oscillation parts of resistivity $\Delta\rho_{xx}$ as a function of $1/(\mu_0 H)$ at various pressures are shown in Supplementary Fig.6b. The fast Fourier transform (FFT) spectra of the QOs reveal four principal frequencies (18, 26, 72 and 92 T) at

ambient pressure as shown in Supplementary Fig.6c. These frequencies remain the same as that at pressure of 0.36 GPa. With the pressure above P1, the QOs damping rapidly and we can hardly extract the reliable frequencies.

The effective mass can be extracted from the temperature dependence of the amplitude of FFT peak using the Lifshitz-Kosevich (LK) formula[4]. The oscillation amplitude at a fixed magnetic field is proportional to the thermal damping factor R_T: $R_T = \frac{\alpha m^* T}{B sinh(\alpha m^* T/B)}$, where α = 14.69 T/K is a constant, B = μ_0H is the magnetic flux density (taking the average value of the field window used for the FFT of QOs), and m* = m/m_e is the cyclotron mass ratio (m_e is the mass of free electron). The extracted fast Fourier transform frequency under various temperatures with pressure of 0.36 GPa are shown in Supplementary Fig.7. The temperature dependence of FFT amplitude of F1 and F2 can be fitted very well with LK formula as shown in the inset of Supplementary Fig.7, and yields effective mass to be 0.025m_e and 0.034m_e for F1 and F2 orbits, respectively. Comparing with the extracted effective mass of 0.028 m_e and 0.031m_e for F1 and F2 at ambient pressure[5], the effective mass does not change much under pressure. The amplitude of the frequencies at 26 and 72 T dramatically increases at 0.36 GPa as shown in Supplementary Fig.6c. The amplitude of the QOs depends on R_T and Dingle damping factor R_D=exp(-αT_Dm*/B), where T_D is the Dingle temperature which is associated with the scattering rate. Since the effect mass does not change much at 0.36 GPa, the enhancement of amplitude can be attributed to the reduction of the scattering rate for the two bands.

Quantum criticality may appear when the CDW transition is totally suppressed. In order to check the possibility of quantum criticality at P2, we replot the low-temperature resistivity as a function of T^2 as shown in Supplementary Fig.8. The low-temperature resistivity above P2 follows T^2 behavior below 35 K indicating the Fermi liquid nature in the normal state, in contrast with the quantum critical behavior. In addition, the residual resistivity does not show a sharp enhancement around P2. For these reasons, our results do not support the quantum criticality at P2. However, ultralow-temperature experiments are still highly required to clarify possible quantum criticality at lower temperature.

The upper critical field can be fitted well by using an effective two-band model[5],

$$a_0[\ln t + U(h)][\ln t + U(\eta h)] + a_1[\ln t + U(h)] + a_2[\ln t + U(\eta h)] = 0$$

where $a_0 = 2(\lambda_{11}\lambda_{22} - \lambda_{12}\lambda_{21})$, $a_1 = 1 + (\lambda_{11} - \lambda_{22})/\lambda_0$, $a_2 = 1 - (\lambda_{11} - \lambda_{22})/\lambda_0$,
$\lambda_0 = [(\lambda_{11} - \lambda_{22})^2 + 4\lambda_{12}\lambda_{21}]^{1/2}$, λ_{11} and λ_{22} are the intraband BCS coupling constants, while λ_{12} and

λ_{21} are the interband BCS coupling constants. The function U(x) = ψ(1/2+x) - ψ (1/2), where ψ is the di-gamma function. $t = T/T_c$, $h = H_{c2}D_1/2\phi_0 T$ and $\eta = D_2/D_1$, where ϕ_0 is the flux quantum and D_n is the electron diffusivity for the nth Fermi-surface sheet. The coherence lengths can be calculated as $\varepsilon_n = \sqrt{D_n/2\pi T_c}$, which are shown in Supplementary Fig.9. Both ε_1 and ε_2 show minimum values around P1 and P2.

Supplementary Fig.10 shows the expanded low-pressure phase diagram with T_c measured by various methods.

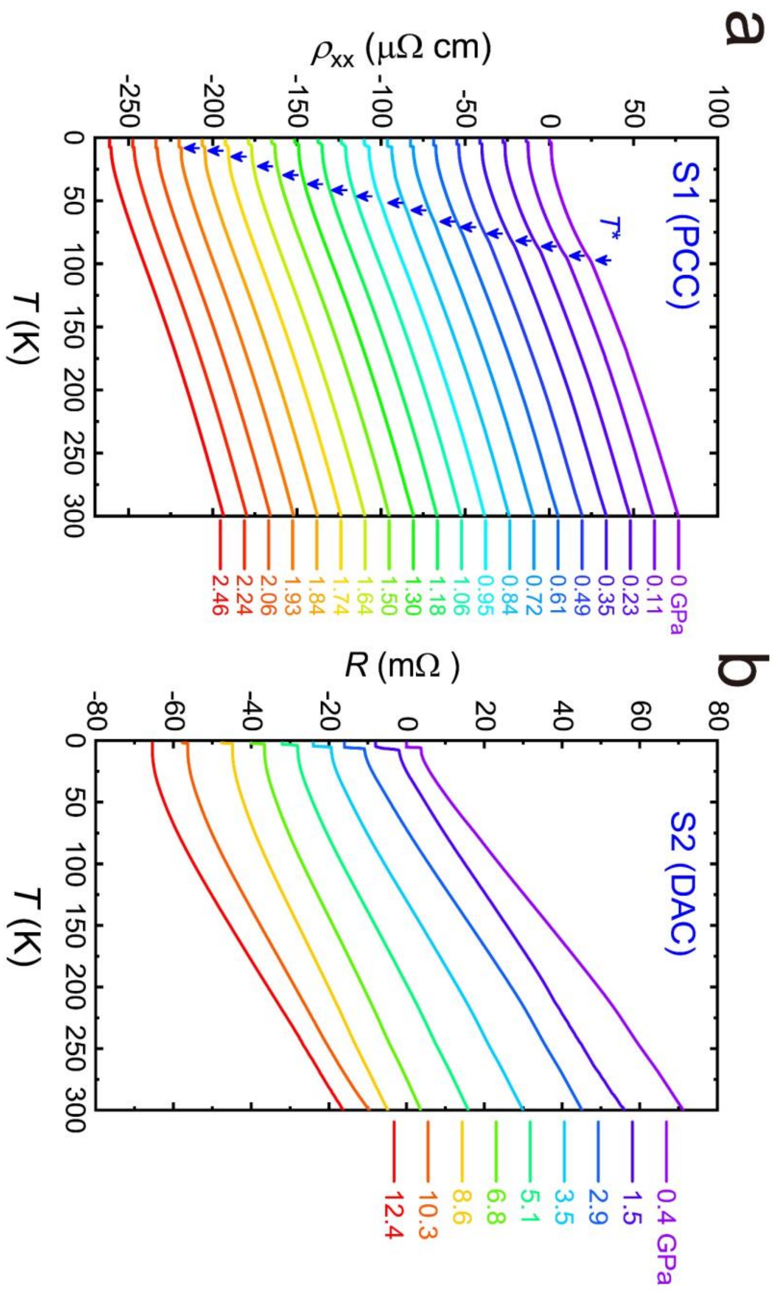

Supplementary Fig.1. (a): Temperature dependence of resistivity in the temperature range from low temperature to room temperature at various pressures for sample 1 measured with PCC. (b): Temperature dependence of resistance in the temperature range from low temperature to room temperature for sample 2 with DAC. All the curves were shifted vertically for clarity.

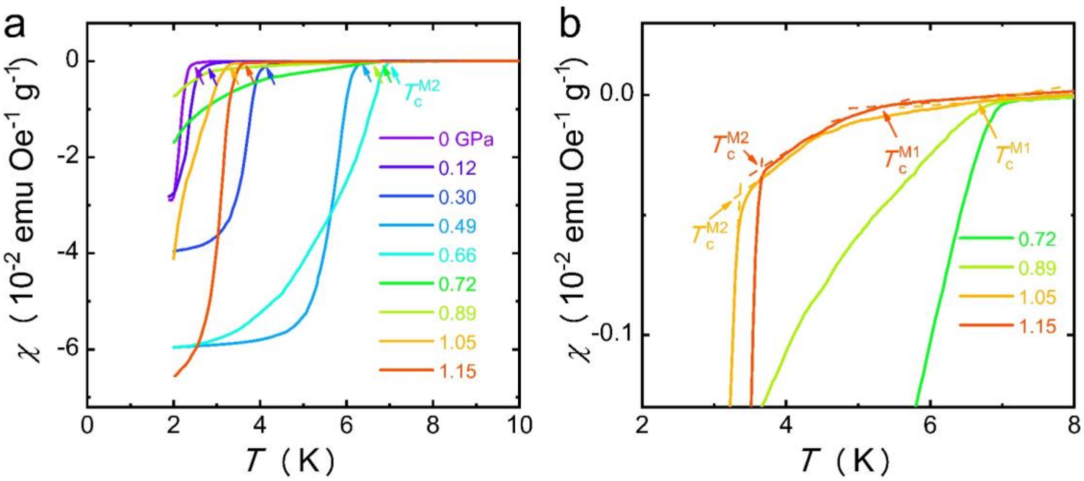

Supplementary Fig.2. (a): High-pressure magnetic susceptibility measurement for sample 3 with PCC. (b): Enlarged area of magnetic susceptibility around T_c with pressure above P1. T_c^{M2} represent the bulk superconducting temperature and T_c^{M1} represents the transition of filamentary superconductivity.

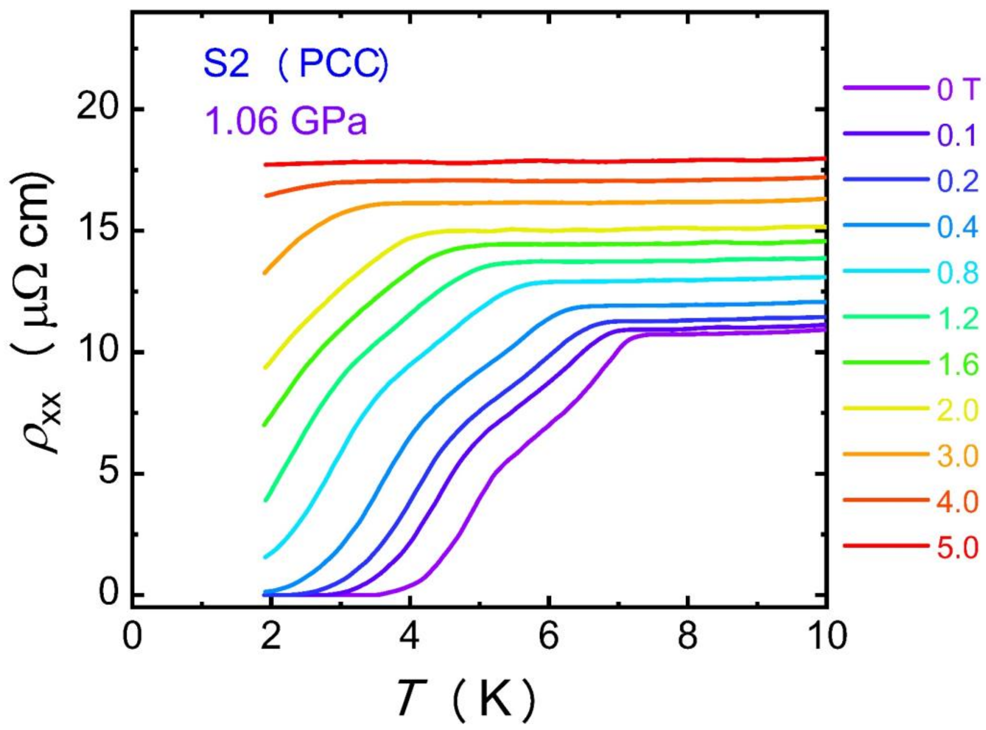

Supplementary Fig.3. Temperature dependence of resistivity under various magnetic fields with pressure of 1.06 GPa.

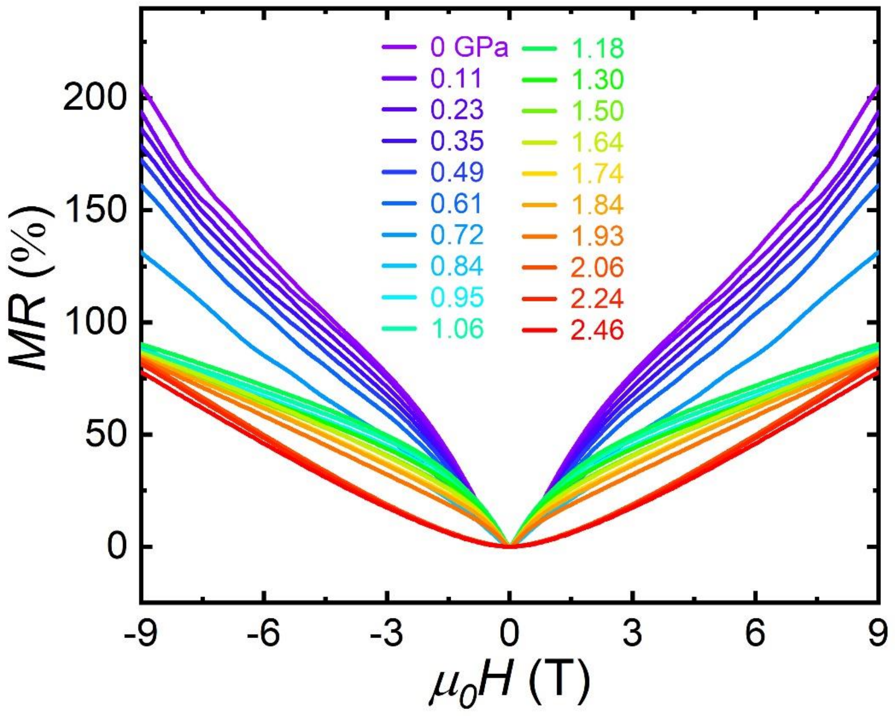

Supplementary Fig.4. Magnetoresistance measured at 10 K under external magnetic field applied along *c* axis and at various pressures for sample 1 with PCC. The magnitude of magnetoresistance suddenly decreases above P1. The low-field magnetoresistance evolves from “V”-shape to “U”-shape at P2.

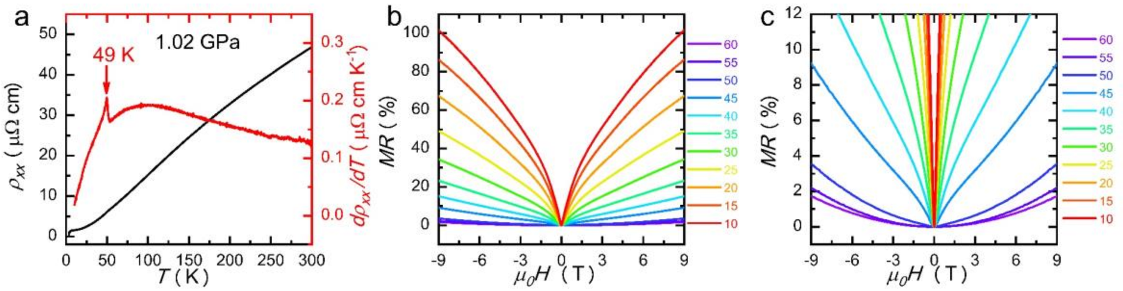

Supplementary Fig.5. (a): Temperature dependences of resistivity and the derivative $d\rho_{xx}/dT$ curve for sample 4 with the pressure at 1.02 GPa. T* is determined to be 49 K. (b) and (c): Magnetoresistance measured at various temperatures with external magnetic field applied along *c* axis. Magnetoresistance suddenly changes around T*.

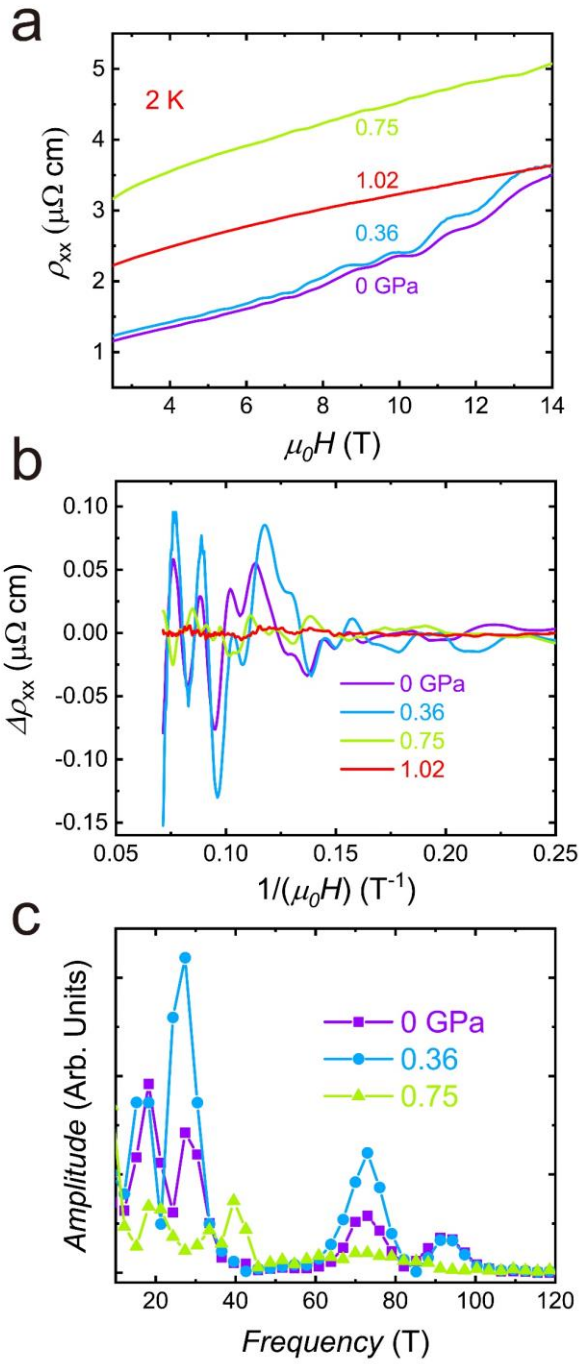

Supplementary Fig.6. (a): The magnetoresistance measured under external magnetic field applied up to 14 T along *c* axis at 2 K and at several pressures for another sample with PCC. (b): The oscillation parts of resistivity $\Delta\rho_{xx}$ after subtracting the background as a function of $1/(\mu_0 H)$ at various pressures. (c): Extracted fast Fourier transform spectra at various pressures.

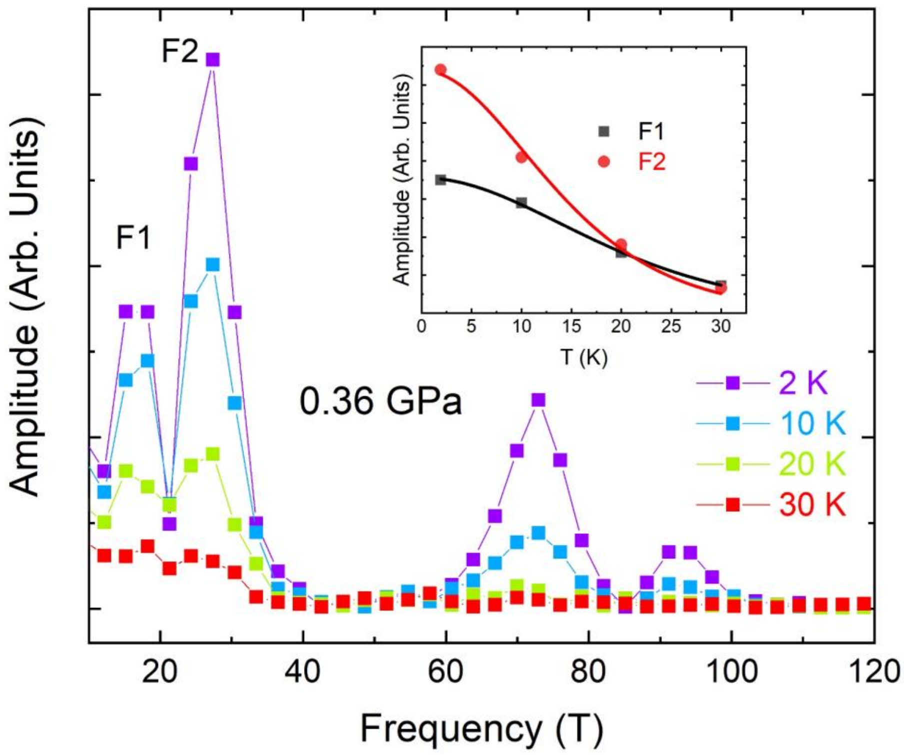

Supplementary Fig.7. Extracted fast Fourier transform frequency of CsV_3Sb_5 measured at various temperatures with pressure of 0.36 GPa. The inset shows the Lifshitz-Kosevich fit of the F1 and F2 orbits to extract the effective mass.

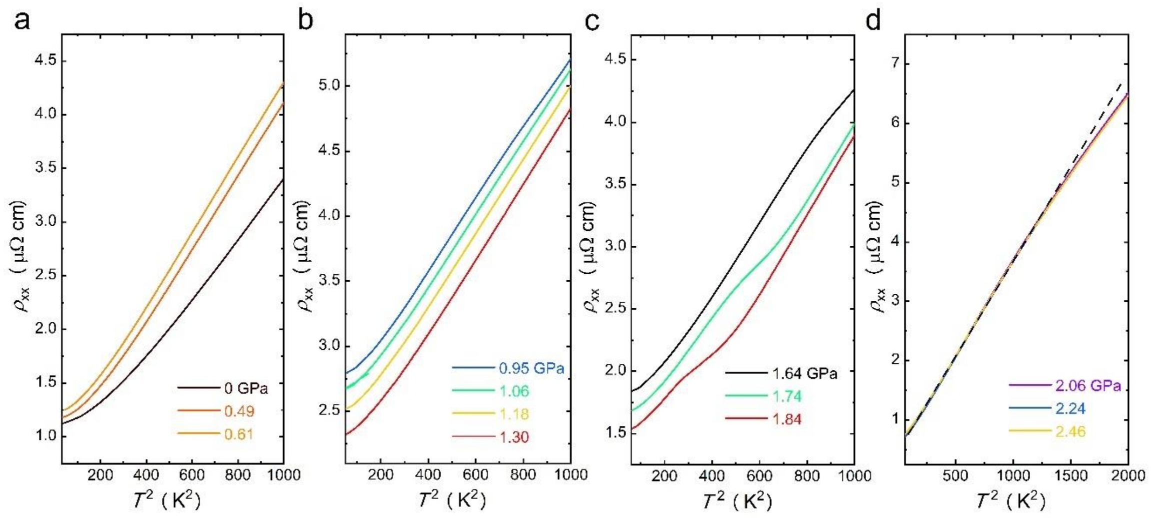

Supplementary Fig.8. Low-temperature resistivity as a function of T^2 under pressure. The resistivity curves above P2 are nearly the same and follow the T^2 behavior below 35 K, indicating the Fermi liquid nature for CsV_3Sb_5 at P2. The anomaly for resistivity between 1.64 and 1.84 GPa is related to the CDW transition.

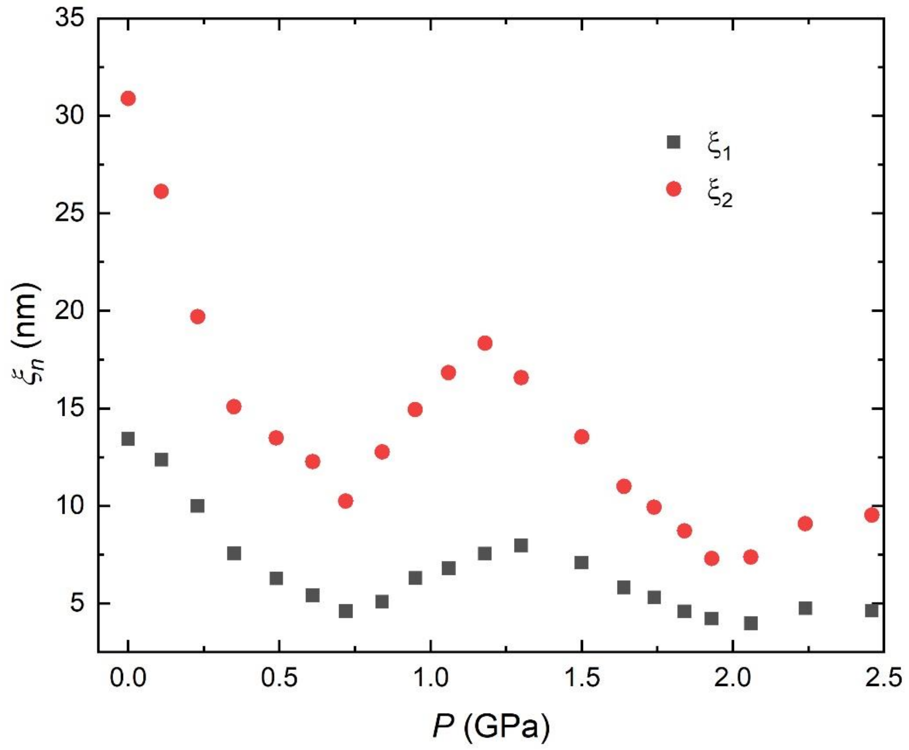

Supplementary Fig.9. Temperature dependence of coherence length ε_1 and ε_2 derived from the two-band model fitting of the upper critical field. Both ε_1 and ε_2 show minimum values around P1 and P2.

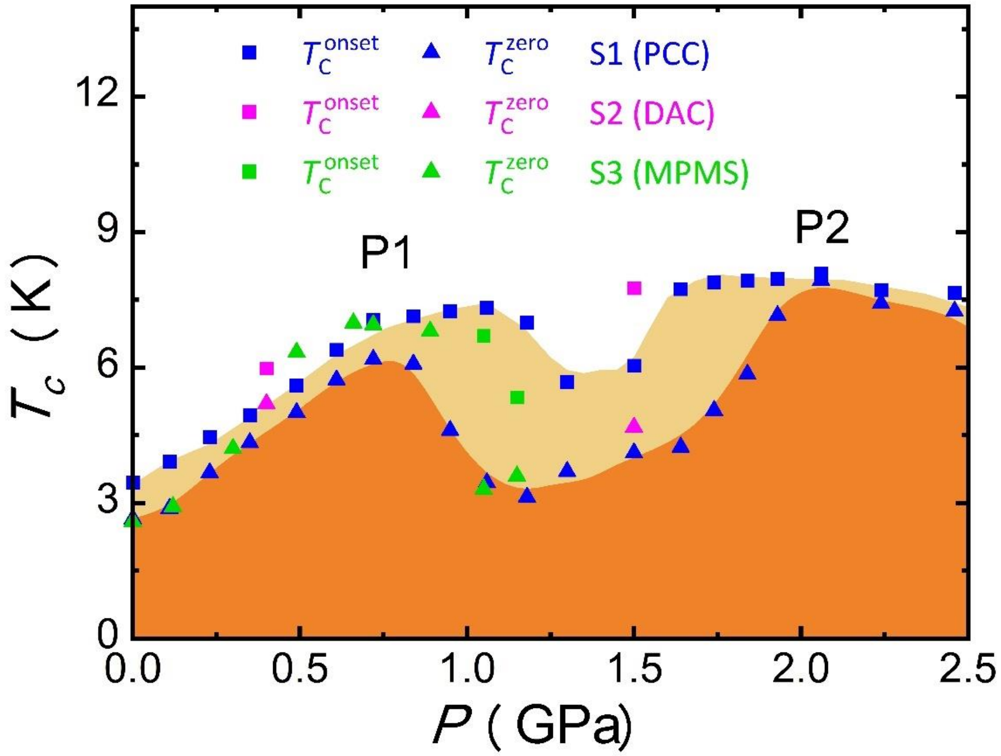

Supplementary Fig.10. Pressure dependence of superconducting transition temperatures T_c^{onset}, T_c^{zero}, T_c^{M1} and T_c^{M2} measured on various samples below 2.5 GPa.

1 Ortiz, B. R. *et al.* CsV_3Sb_5: A Z_2 Topological Kagome Metal with a Superconducting Ground State. *Physical Review Letters* **125**, 247002 (2020).

2 Abrikosov, A. A. Quantum magnetoresistance. *Physical Review B* **58**, 2788-2794 (1998).

3 Feng, Y. *et al.* Linear magnetoresistance in the low-field limit in density-wave materials. *Proceedings of the National Academy of Sciences* **116**, 11201-11206 (2019).

4 Shoenberg, D. *Magnetic Oscillations in Metals*. (Cambridge University Press, 1984).

5 Yu, F. H. *et al.* Concurrence of anomalous Hall effect and charge density wave in a superconducting topological kagome metal. *arXiv:2102.10987* (2021).

6 Gurevich, A. Enhancement of the upper critical field by nonmagnetic impurities in dirty two-gap superconductors. *Physical Review B* **67**, 184515 (2003).
